# Reliability of artificial intelligence algorithms in automated age estimation using orthopantomograms: A scoping review

**DOI:** 10.1177/20552076251390556

**Published:** 2025-11-12

**Authors:** Spoorti Kulkarni, Sudeendra Prabhu, Andrew Jeyabose, Vikram Palimar

**Affiliations:** 1 Department of Oral and Maxillofacial Pathology and Oral Microbiology, Manipal College of Dental Sciences, Manipal, Manipal Academy of Higher Education, Manipal, Karnataka, India; 2 Department of Oral Pathology and Microbiology, Centre for Forensic Odontology, Yenepoya Dental College, Yenepoya deemed to be university, Mangalore, Karnataka, India; 3 Department of Computer Science & Engineering, Manipal Institute of Technology, Manipal Academy of Higher Education, Manipal, Karnataka, India; 4 Department of Neurology, School of Medicine, University of North Carolina at Chapel Hill, NC, USA; 5 Department of Forensic Medicine and Toxicology, Kasturba Medical College, Manipal, Manipal Academy of Higher Education, Manipal, Karnataka, India

**Keywords:** Age estimation, forensic odontology, machine learning, deep learning

## Abstract

**Background:**

This study aims to evaluate the efficiency of AI (artificial intelligence) algorithms for automated age estimation using orthopantomograms (OPGs) and to determine whether these models can effectively replace conventional age estimation techniques.

**Method:**

Three independent literature searches were conducted in PubMed, Scopus, and Embase. Studies published in the English language were considered, focusing on age estimation using AI. A total of 1519 articles were screened, and 24 articles were included in the study. The data was extracted in a standardized, predefined manner. After finalizing the search, the data collected was tabulated, interpreted, and verified. The selected studies were analyzed for methodological rigor, algorithmic performance, and comparative effectiveness against traditional age estimation methods.

**Results:**

AI-based models, especially deep learning architectures like convolutional neural networks, EfficientNet, DenseNet, and hybrid models such as Age-Net, demonstrated superior accuracy, precision, and reliability compared to traditional age estimation methods. These AI-driven models show promising results in reducing human error, increasing efficiency, and enhancing forensic and clinical decision-making.

**Conclusion:**

AI-driven age estimation using OPGs represents a transformative advancement with considerable forensic and clinical potential. Although these AI models may not yet fully replace conventional techniques, they offer a substantial value as complementary tools, improving both accuracy and operational efficiency. To foster wider adoption and improve reliability, ongoing research and the development of standardized protocols are essential for integrating these methods into forensic odontology and related fields.

## Introduction

The advent of artificial intelligence (AI) has revolutionized various fields, including healthcare and dentistry. One of the promising applications of AI is in the realm of age estimation, particularly through the analysis of orthopantomograms (OPGs). Accurate age estimation is crucial in various contexts, such as forensic science, anthropology, and pediatric dentistry, where chronological age may influence treatment decisions and legal proceedings.^1^

In India, the growing population and increasing reliance on digital health technologies necessitate the development of reliable AI algorithms for age estimation. Traditional methods of age determination, which often rely on manual assessment by dental professionals, can be time-consuming and subjective. While AI has been extensively adopted in various medical fields, its integration into dentistry, particularly in forensic odontology, is still in its preliminary stages. The integration of AI into this area promises enhanced accuracy and efficiency, potentially reducing human error and improving diagnostic outcomes.^2,3^

AI is rapidly transforming the field of dentistry by automating diagnostic, analytical, and decision-making capability.^4,5^ Digital dentistry (digital imaging, computer-aided design and computer-aided manufacturing) is already reshaping clinical workflows such as auto-designing dental restorations, optimizing implant placement, and analyzing datasets to predict outcomes, personalize treatment, and reduce the risk.^6^ In forensic odontology, AI has significant potential to enhance human identification and age estimation, which are crucial aspects of forensic practice. AI technologies, particularly deep learning (DL) and convolutional neural networks (CNNs), can analyze panoramic radiographs, intraoral scans, and cone beam computed tomography images to predict dental maturity and chronological age. Automated systems have demonstrated accuracy in staging third molar development, which is an important tool for determining legal age thresholds in forensic cases. Additionally, AI can quantify pulp–tooth ratios (PTRs) and extract radiomic features, thus improving the accuracy of adult age estimation. Beyond age estimation, AI can also classify dental structures for comparative identification, perform bite-mark analysis, and recognize unique dental patterns.^4,5^ DL approaches can also be used to automate and enhance the detection of dental issues in OPGs, such as broken roots, periodontally compromised teeth, and the Kennedy classification of partially edentulous arches.^7^ Despite the potential benefits, the reliability of AI algorithms in automated age estimation remains underexplored. Specialized postgraduate programs are crucial to prepare the next generation of dentists who are equally skilled in traditional clinical dentistry and digital/AI-driven dentistry.^5^ A thorough review is warranted to evaluate existing literature, identify gaps in the current understanding, and assess the applicability of AI-driven techniques.

The main research question of this scoping review was to assess the latest developments in using AI for age estimation from OPGs, and to determine if these models demonstrate an advantage over conventional techniques by achieving lower mean absolute error (MAE) and improved diagnostic performance. Thus, the main objective of this paper was to determine the efficiency of AI-driven algorithms derived by the automated age estimation process using OPGs and to validate the potential of automated age estimation, utilizing OPGs, and to assess whether AI-based models can effectively replace conventional techniques of age estimation using OPGs. This scoping review offers a unique synthesis of 24 original studies focused on AI-based age estimation using OPGs, building on existing reviews of AI applications in forensic and dentistry. It critically compares various AI models with traditional techniques. The findings indicate that AI consistently achieves lower MAE, greater accuracy, and less subjectivity compared to manual assessments. The paper also highlights the forensic applications of AI in determining legal age thresholds (14, 16, and 18 years), disaster victim identification (DVI), and human identification tasks. The study evaluates the limitations of algorithms, focusing on issues such as data scarcity, imbalanced datasets, and the lack of generalizability across diverse populations. It also emphasizes the need for standardized protocols and validation frameworks.

## Methods

### Search strategy

Our study followed the preferred reporting items for systematic reviews and meta-analyses extension for scoping reviews framework, a thorough digital search for data using reliable databases, including PubMed, Scopus, and Embase was done.^8^ To find relevant articles in these electronic databases, we employed a variety of key terms: “Artificial Intelligence,” “Machine learning,” “Deep Learning,” “Deep neural networks,” “Convolutional neural networks,” “Age estimation,” “Forensic dentistry,” and “Forensic odontology.” We also utilized Boolean operators (AND, OR) and applied filters for articles written in English. Table 1 outlines the detailed search strategy used for this scoping review.

**Table 1.** Summary of search terms, medical subject headings terms, and Boolean operators/filters applied.

Database	Keywords used	Medical subject headings terms	Boolean operators/filters applied
PubMed	“Artificial Intelligence,” “Machine learning,” “Deep Learning,” “Deep neural networks,” “Convolutional neural networks,” “Age estimation,” “Forensic dentistry,” “Forensic odontology”	“Intelligence, artificial,” “computer reasoning,” “reasoning, computer,” “machine intelligence” “Intelligence, machine,” “computational intelligence,” “Intelligence, computational,” “computer vision systems,” “computer vision system,” “vision systems,” “Knowledge acquisition (computer),” “knowledge representation (computer),” “learning, machine,” “transfer learning,” “learning, transfer,” “learning, deep,” “hierarchical learning,” “learning, hierarchical” “Convolutional neural network,” “neural network,” “dentistry,” “forensics”	AND, OR Publication date: 10 years (2015–2025) Text availability: Full text published in English language. Type of study: original studies (observational)
Scopus	The search stratergy was same as that of Pubmed	“Artificial Intelligence,” “machine learning,” “deep learning,” “deep neural networks,” “convolutional neural networks,” “age estimation,” “forensic dentistry,” “forensic odontology”	AND, OR
Embase	The search stratergy was same as that of Pubmed	Emtree terms: “ambient intelligence,” “artificial general intelligence,” “artificial intelligence agent,” “automated reasoning,” “generative artificial intelligence,” “Artificial neural network”; “automated pattern recognition” “feature learning (machine learning),” “Pattern analysis (machine learning),” “perceptron predictive learning (machine learning),” “Semi supervised machine learning,” “Supervised machine learning,” “transfer learning (machine learning),” “unsupervised machine learning,” “dental age determination”	AND, OR

### Eligibility criteria

#### Inclusion criteria

1. *Study design*: Original research articles (observational or retrospective) evaluating age estimation.
2. *Population*: OPGs were used as the primary radiographic data source.
3. *Intervention*: Use of AI, machine learning (ML), or DL models for age estimation.
4. *Comparisons*: Studies comparing AI-based models with traditional techniques and solely AI models were included.
5. *Outcomes*: Measured in terms of at least one performance metric, including accuracy, sensitivity, specificity, precision, recall, MAE, root mean-squared error, coefficient of determination (*R*^2^), or standard error of estimate.
6. *Publication type and language*: Full-text, peer-reviewed articles published in English.

#### Exclusion criteria

1. Nonoriginal works (reviews, systematic reviews, meta-analyses, commentaries, technical notes, letters to the editor).
2. Case reports, pilot studies, and conference abstracts.
3. Articles not available as full text or published in languages other than English.
4. Studies not directly involving AI/ML/DL algorithms for age estimation.
5. Research without human OPG data (e.g. animal studies or other imaging modalities).
6. Studies with insufficient methodological details or without clearly reported performance outcomes.

### Data charting process

The initial screening of titles and abstracts, along with the removal of duplicates in an Excel sheet (version 1.19.4), was conducted based on the established inclusion and exclusion criteria. Data charting was performed independently by two reviewers (S.K. and S.P.), with subsequent verification by the senior reviewer (V.P.). Any discrepancies were resolved by a third reviewer (A.J.) to ensure the accuracy and reliability of the extracted information. The Excel sheet recorded details such as the authors, study population, year of publication, and country of study. The intervention focused on the application of AI models in age estimation, along with various conventional techniques used, as well as the outcomes and limitations of the studies were evaluated.

## Results

### Selection of the source of evidence

**Figure 1.**
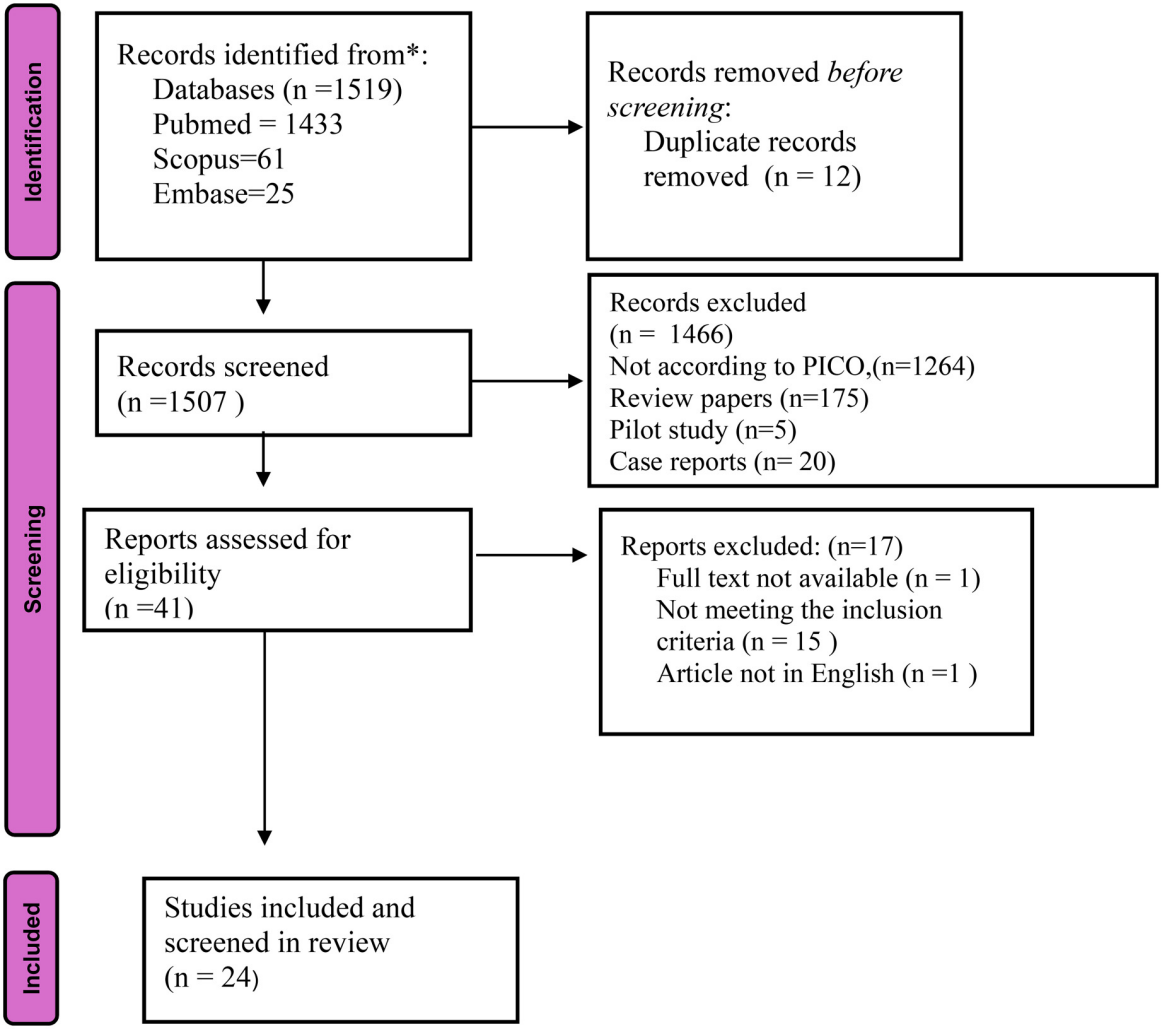
Total number of articles screened in the databases.

A total of 1519 records were retrieved across PubMed (*n* = 1433), Scopus (*n* = 61), and Embase (*n* = 25). After removing 12 duplicates, 1507 unique records were screened. Of these, 1466 articles were excluded at the title/abstract stage (1264 did not align with population intervention comparison outcome, 175 review papers, 5 pilot studies, 20 case reports, and 2 commentaries/technical notes). Forty-one full-text articles were assessed for eligibility, and 17 were excluded (1 due to unavailability of full text, 15 not meeting strict inclusion criteria, and 1 not in English). Ultimately, 24 studies met the inclusion criteria and were included in this scoping review. Figure 1 illustrates the search strategy employed in this scoping review.

### Characteristics of sources of evidence

The included studies varied widely in population with sample size ranging from 554 to 50,000 OPGs and geographical settings (South Korea, India, Turkey, Belgium, Brazil, Thailand, China, Germany, Malaysia). Both conventional forensic methods (Demirjian's, Kvaal's, Gustafson's) and AI models (CNNs, EfficientNet, DenseNet, visual geometry group [VGG], Inception, MobileNet, Age-Net, transfer learning approaches, and XGBoost) were employed. The main outcome of our research question was measured in terms of MAE, accuracy, area under the curve (AUC), and *F*1 scores.

### Results of individual sources of evidence

*AI for age estimation using developmental stages (Demirjian's method)*: YOLOv5, U-Net, and EfficientNet achieved mean average precision (mAP) of 0.995 and segmentation accuracy of 0.978.^9^ DenseNet-201 produced an MAE of 0.73 years, considerably lower than traditional Demirjian staging.^10^ Studies highlighted that the mandibular molars, especially second molars, as the most reliable markers for CNN-based age estimation. Artificial neural network-multilayer perceptron (ANN-MLP) models in specific populations minimized the difference between chronological age and dental age.^11,12^ These findings demonstrate that AI markedly improves accuracy compared with Demirjian's conventional approach.

*AI for age estimation based on third molar development*: DenseNet-121 with transfer learning achieved AUC values of 0.94 (males) and 0.83 (females) at the 18-year threshold. CNNs applied to large datasets classified individuals under 18 versus over 18 with accuracies of 87–88%.^13,14^ Root length-based DL models achieved 87.2% accuracy, slightly outperforming support vector machines (SVMs).^15^ Quadratic regression with Demirjian staging confirmed a 100% probability of being over 18 once mandibular third molars reached stage H.^16^

*AI for PTRs*: InceptionV4-based CNN models analyzing 12,827 radiographs achieved an MAE of 3.1 years (*R*^2^ = 95.5%).^17^ ML (XGBoost) reduced MAE to 4.65 versus 5.68 years with Kvaal's method.^18^

*AI using Gustafson's criteria*: Partial least squares (PLS) regression achieved an MAE of 4.15 years in males, while support vector regressor (SVR) achieved 3.81 years in females.^19^

*AI in human identification, gender determination*: Beyond age estimation, the role of AI is extended to tasks such as tooth identification, sex determination, and DVI. CNNs trained on large datasets of dental radiographs have achieved accuracies between 87% and 99% for tooth type classification, approximately 76% for sex determination, and median age estimation errors around 4.9 years. Notably, the predictive accuracy was higher for healthy, untreated teeth compared with those affected by restorations or pathology, underscoring the influence of dental alterations on model reliability.^20^

Transfer learning approaches, such as AlexNet-based models, further enhanced human identification by localizing and classifying, reaching 95% accuracy for tooth classification and 100% accuracy in distinguishing upper versus lower jaws.^21^ DL model like DENT-net achieved strong performance in forensic identification, reporting Rank-1 identification accuracy of 85%, with high precision (0.90), recall (0.95), and *F*1 score (0.92), while also operating at rapid processing speeds suitable for large-scale forensic casework.^22^ EfficientDet-D3 CNN architectures showed high accuracy in detecting dental features in mass disaster victims, with average precision of 99.1% and recall of 99.6%, though recall was lower for prostheses (84.3%) and root canal-treated teeth (89.2%).^23^ Additionally, CNN-based models trained on broader datasets of panoramic radiographs demonstrated age group classification accuracies of 53.8% within ±5 years, 95.1% within ±15 years, and 99.6% within ±25 years, with performance again favoring healthy dentitions.^24^

*Comparative performance of AI models for age estimation*: With hyperparameter optimization, EfficientNet-B4 achieved the lowest MAE of 0.562 years, while DenseNet-201 and MobileNet V3 demonstrated MAEs of 0.58 and 1.78 years, respectively.^25^ Large-scale CNN applications also reported strong results; a standardized CNN trained on 50,000 OPGs attained MAEs of 2.76 years (postmortem) and 3.26 years (antemortem) while significantly reducing processing time. Other models showed variable performance depending on dataset size and age group.^26–28^ DentAge, a DL system trained on over 21,000 radiographs, achieved an overall MAE of 3.12 years, with its best performance in adolescents (MAE 1.94 years) but limited accuracy in the elderly (MAE 13.40 years).^29^ Similarly, EfficientNet-B5 performed best in younger cohorts (12–21 years), achieving an MAE of 2.8 years. Heuristic grouping strategies, such as applying a ±3-year tolerance, improved performance metrics for WideResNet and DenseNet architectures.^30^

Hybrid models integrating DL with ML classifiers, such as Age-Net (EfficientNetB0 + SVM), achieved an AUC of 0.970 and an accuracy of 76.41%.^31^ The studies comparing VGG19, ResNet152, SqueezeNet, and EfficientNet showed that VGG19 consistently outperformed others in simpler binary tasks (92.95% accuracy) but struggled in more complex classifications.^32^ Table 2 provides an overview of the data extraction process and key characteristics from the studies reviewed.^9–32^

**Table 2.** Summary of data extraction from various studies.

Authors	Year/population	Sample size	AI vs. conventional technique/other AI models	AI-based models	Study results	Outcome	Limitations
Ong et al. ^9^	2024/South Korea	5133	Demirjian's method	Deep learning models; YOLOv5 model was used for detection, U-Net for segmentation, and EfficientNet for classification of the teeth	mAP 0.995 for detection and an accuracy of 0.978 for segmentation	Best classification accuracy was observed in the molar model (*F*1 score = 90.81%)	Low accuracy in the anterior teeth which could be due to low-resolution panoramic radiographs and patient positioning errors
Sivri et al.^10^	2024/Turkey	5898	Demirjian's method	CNN -DenseNet-201	MAE 0.73 years	CNN outperformed conventional method	Only radiographs of individuals aged 4 to 17 years were considered
Matthijs et al.^11^	2024/Germany	1639	Ten-stage modified Demirjian staging technique	CNN-DenseNet	MAE 0.71	Mandibular molars gave better results and can be used for automated age estimation compared to incisors	Distinguishing between closely related developmental stages, could affect the precision of age estimation
Bunyarit et al.^12^	2022/Malaysian Indian children	1015	Chaillet and Demirjian's modified eight-tooth method	ANN-MLP	Mean difference: 1.68 years for boys and 2.56 years for girls	ANN-MLP provides better maturity scores	Variability in dental development due to genetic and environmental factors may contribute to discrepancies in age estimation
Franco et al.^13^	2024/Brazil	11,640	Deep learning models	CNN-DenseNet-121	AUC 0.94 (males), 0.83 (females)	AI improves forensic age classification	The complexity of third molar morphology and surrounding structures posed difficulties in image annotation, potentially affecting the model's performance
Murray et al.^14^	2023/Brazil	4003	3rd molar development in age estimation	CNN	High accuracy in under/over 18 classification. AUC of 0.87, 88% accuracy for subjects above 18 years and 87% for those under 18 years	CNN models can be used for age classification, above/below 18 years	The model was trained to classify individuals as either under or over 18 years old. Its performance for age estimation outside this specific threshold remains untested. Dental development can vary across populations, so the model's accuracy might differ when applied to groups not included in the training data
Patil et al.^15^	2023/India	1000	ML vs. DL in age estimation using mesial root length	DLM (fully connected neural network), SVM	Accuracy 87.2% (2-Class), 66% (3-Class), 42.8% (5-Class)	DLM outperformed traditional MLM, with the mesial root length of the right third molar being a significant predictor of age	The reliance on root length as a sole predictor of age may overlook other contributing factors, necessitating a more comprehensive approach in future studies
Duangto et al.^16^	2017/Thailand	1867	3rd molar development according to Demirjian's method	Quadratic regression was used to develop age prediction models	*R* = 0.945 for tooth 38 in males	Age prediction models with low error values were developed for age estimation in Thai samples which was useful to establish a cut off of above or below 18 years, based on 3rd molar development	The development of third molars can be highly variable among individuals, and factors such as impaction or agenesis would affect the model
Oliveira et al.^17^	2024/Brazil	12,827	Deep learning models	CNN: InceptionV4	MAE of 3.1 years, RA, *A*^2^ = 95.5%	InceptionV4 architecture, combined with effective data augmentation strategies, significantly improved the model's performance	Distortion in panoramic radiographs can impact the model's accuracy
Pereira de Sousa et al.^18^	2023/Brazil	554	Kvaal method	Machine learning	XGBoosting regression algorithm MAE 4.65 years (ML), 5.68 (Kvaal)	ML enhances accuracy over Kvaal’s method	Smaller sample size and population was not evenly divided among different age groups
Dai et al.^19^	2024/Southwest China	851	Gustafson's method	Machine learning; PLS, SVR, GBR	MAE -4.151 (males), 3.806 (females)	PLS is best for males, SVR is best for females	Imbalanced age distribution
Milošević et al.^20^	2022/Zagreb	2899	Deep learning models	CNN	76.41% accuracy (sex), MAE 4.94 years	Requirement of diverse datasets	Alterations in the tooth can affect the models accuracy
Sathya and Neelaveni^21^	2020/India	3159	Deep learning models	Transfer learning using the AlexNet architecture	100% accuracy in classifying teeth into upper or lower jaw, overall accuracy of 95% for classifying teeth into molar, premolar, canine, or incisor	The proposed three-stage transfer learning approach for human identification using dental traits has shown effective results in terms of accuracy and precision	Blurred images could affect the identification process
Fan et al.^22^	2020/China	15,369 from 6000 individuals	Forensic identification	DENT-Net (customized CNN)	The recall, precision, and *F*1 score of the DENT-Net were 0.95, 0.90, and 0.92, respectively. The AUC for the DENT-Net was 0.996	Due to its accuracy in identification of tooth in less span of time (33.03 ms) makes it useful in mass disaster cases and criminal identification	The DENT-Net was not optimized for human identification on mixed dentition, posing a challenge for manual comparison. Teeth overlap, imaging positions, and poor quality of dental images, pose a problem
Choi et al.^23^	2022/Korea	1638	Deep learning models	Deep learning model (CNN) modified by EfficientDet-D3	Natural teeth: precision 99.1%, recall 99.6%. Prostheses: precision 80.6%, recall 84.3%. Treated teeth: precision 81.2%, recall 89.2%. Implants: precision 96.8%, recall 98.1%	This AI model is effective in accurately identifying and classifying dental features on dental panoramic radiographys	The model exhibited lower precision and recall rates for detecting prostheses compared to other categories, suggesting a need for further refinement in this area
Kim et al.^24^	2023/Korea	10,023	Forensic identification	DNN	Accuracies of 53.846% (±5 years), 95.121% (±15 years), and 99.58% (±25 years)	The model is more reliable when broader age ranges are acceptable	Misclassifications due to implants and treated teeth
Büyükçakır et al.^25^	2024/Belgium	3896	Demirjian's method	CNN- EfficientNet-B4, DenseNet-201, MobileNet V3	EfficientNet: MAE of 0.562 years. DenseNet-201: MAE of 0.58, MobileNet V3: MAE of 1.78	Hyperparameter optimization significantly enhances the performance of deep learning models for dental age estimation from OPGs	Data scarcity and the need for vigorous training methodologies
Kim et al.^26^	2023/South Korea	2760 of 746 OPGs	Deep learning models	Deep Learning technique, VGG16	Rank-1 accuracy: 80.56% without augmentation, with augmentation: 82.84%. Rank-3 accuracy, without augmentation: 86.86%, with augmentation 89.14%. Rank-5 accuracy without augmentation: 88.61%, with augmentation: 92.23%	Fully automated human identification with high accuracy has fast identification process (3.2 s per image), aiding forensic and disaster victim identification	Image distortion could affect the study results
Bussaban et al.^27^	2023/Thailand	785	Deep learning models	VGG19, ResNet152, SqueezeNet, EfficientNet	VGG19 best: 92.95% (2-Class), ResNet -80% (4-Class), SqueezeNet-75.66% (6-Class), EfficientNet-69.11% (12-Class)	Deep learning enhances accuracy	Dataset size was a challenge
Heinrich^28^	2024/Germany	50,000	Deep learning models	CNN	MAE 2.76 years (postmortem), 3.26 years (antemortem)	CNNs streamline identification with high reliability	A smaller sample size for post-mortem cases
Bizjak and Robič^29^	2024/Slovenia	21,007	Deep learning models	DentAge (deep learning model)	MAE 3.12 years, for 10 to 20 years, MAE 13.4 years for 90–100 years	DentAge shows high accuracy with transfer learning	MAE was higher for subjects over 95 years old, indicating limitations in accurately predicting age for this demographic
Kahm et al.^30^	2023/South Korea	27,877 images	Deep learning models in tooth development, eruption, and mineralization stages	WRN, DN	*F*1 score: 0.6583 (DN), 0.6437 (WRN)	Type 2 grouping (heuristic grouping), especially when incorporating a ±3-year deviation, yielded higher accuracy and *F*1 scores compared to type 1	External validation would increase the robustness of the study
Baydogan et al.^31^	2023/Turkey	933	Deep learning models and hybrid models	Age-Net (hybrid AI model)	AUC 0.970	EfficientNetB-SVM combination of these models yielded better results	The dataset was restricted to a particular age group and small sample size
Mu and Gang^32^	2021/China	3000	Deep learning models	ResNet, EfficientNet, VGGNet, DenseNet	MAE 2.83, RMSE 4.59 (EfficientNet-B5)	Transfer learning model EfficientNet-B5 improves age accuracy	With a relatively small dataset, there's a risk that the models may over fit

AI: artificial intelligence; mAP: mean average precision; CNN: convolutional neural network; ANN-MLP: artificial neural network-multilayer perceptron; AUC: area under the curve; ML: machine learning; DL: deep learning; DLM: deep learning model; SVM: support vector machine; MLM: machine learning model; PLS: partial least squares; SVR: support vector regressor; GBR: gradient boosting regression; DNN: deep neural network; OPG: orthopantomogram; WRN: WiseResNet; RMSE: root mean-squared error.

## Discussion

The integration of AI into dental radiology has shown significant promise in advancing clinical applications, particularly in age estimation, identification, and classification tasks. This scoping review demonstrates that AI-based models, especially CNNs, DenseNet, and EfficientNet architectures, achieve significantly higher accuracy in dental age estimation compared to traditional techniques. Several studies reported significantly lower MAE values, highlighting the precision of DL approaches in forensic applications. However, limitations such as a lack of dataset diversity, the need for external validation, and insufficient methodological transparency hinder the immediate application of these results in practice. The studies presented in this paper emphasize the potential and challenges of using AI models in the field of forensics.

### Effectiveness of AI in automated age estimation using developmental stages of teeth in children and adolescents (Demirjian's method)

AI-based models have shown notable advancements over Demirjian's traditional method in estimating dental age in children and adolescents. Ong et al.^9^ developed a fully automated staging system on 5133 panoramic radiographs that integrated detection (YOLOv5), segmentation (U-Net), and classification (EfficientNet). The system achieved an mAP of 0.995 for detection and 0.978 accuracy for segmentation, while classification performance varied by tooth type, with the molar model yielding the highest *F*1 scores. Thus, the results highlight the ability of DL approaches to effectively replicate and, in some cases, enhance the staging reliability of Demirjian's technique. However, limitations such as low-resolution images, patient positioning errors, and imbalanced datasets were identified as critical barriers to achieving uniformly high accuracy. Also limited number of samples for early developmental stages was one of the limitations, as panoramic radiographs are not routinely taken at a young age, indicating a need for studies with larger sample sizes. Adding to these findings, Sivri et al.^10^ conducted a comparative study on 5898 pediatric radiographs (ages 4–17 years) and reported an MAE of 0.73 years using DenseNet-201, which was considerably lower than values typically achieved with the traditional Demirjian approach. This reinforces the evidence that CNNs can minimize prediction errors and provide greater consistency compared with conventional staging methods.

Another study by Matthijs et al.^11^ explored the application of the DenseNet-201 model for automated age estimation using 1639 panoramic radiographs. Their approach focused on five mandibular teeth (central incisor, canine, first premolar, first molar, and third molar), wherein a 10-stage modified Demirjian technique for staging was applied. The model's performance varied across tooth types, with the left mandibular second molar (tooth 37) showing the highest accuracy (MAE = 0.71), whereas the left mandibular central incisor (tooth 31) presented greater challenges. These findings highlight that mandibular molars, particularly second molars, offer more reliable developmental markers for CNN-based age estimation. A study by Bunyarit et al.^12^ in the Malaysian population investigated the applicability of Chaillet and Demirjian's modified eight-tooth method for dental age estimation among Malaysian Indian children and adolescents aged 5.00–17.99 years. A total of 1015 dental panoramic radiographs was analyzed. The study employed an ANN-MLP approach to develop new dental maturity scores for Malaysian Indian children and adolescents. The conventional method gave a difference between the chronological age and dental age of approximately 2.09 years in boys and 2.79 years in girls across all age groups. The ANN-MLP model produced a new prediction formula, resulting in a mean difference of approximately 0.035 ± 0.84 years for boys and 0.048 ± 0.93 years for girls, indicating enhanced accuracy. Variability in dental development due to genetic and environmental factors may also contribute to discrepancies in age estimation.

### Effectiveness of AI in automated age estimation based on third molar development

Several studies have emphasized the potential of AI in improving the accuracy of age estimation through third molar assessment. Franco et al.^13^ investigated third molar development around the legal thresholds of 14, 16, and 18 years using a large dataset of 11,640 panoramic radiographs. Employing DenseNet-121 with transfer learning, their model achieved strong performance, with AUC values of 0.94 for males and 0.83 for females at the 18-year threshold, indicating its potential forensic utility. Murray et al.^14^ also applied CNNs to third molar development for determining the legal age of majority. Using 4003 panoramic radiographs, the CNN effectively classified individuals as “under-18” or “over-18” with high predictive accuracy. This study emphasizes the legal and judicial implications of using AI tools, especially in enhancing courtroom decision-making. Patil et al.^15^ assessed age estimation in an Indian population by applying a fully connected DL model based on second and third molar root lengths. The DL approach yielded higher predictive accuracy (87.2% for 2-Class classification) compared with traditional ML models such as SVM (86.4%). However, classification accuracy diminished for more complex stratifications (3-Class: 66%, 5-Class: 42.8%), suggesting that root length alone may not capture the full variability of dental maturation. Similarly, Duangto et al.^16^ employed quadratic regression based on Demirjian staging of third molars in a Thai population (*n* = 1867). Their results demonstrated low error values and found that once mandibular third molars reached stage H, the probability of being over 18 years was 100% in both sexes. However, the limited number of younger participants reduced the strength of conclusions for early developmental stages.

Overall, these findings indicate that AI models, especially CNNs and transfer learning architectures, are very effective at using third molar development for legal age determinations. However, differences among populations, sex variations, and dependence on single indicators like root length remain challenges that future research needs to address to improve broad applicability and forensic use.

### Effectiveness of AI in automated age estimation based on PTR

The application of AI in PTR analysis has shown promising improvements over conventional methods. Oliveira et al.^17^ evaluated a large dataset of 12,827 dental images from Brazilian patients using an InceptionV4-based CNN model that incorporated radiological features such as pulp chamber dimensions and permanent tooth calcification stages. Their model demonstrated strong predictive performance, with an MAE of 3.1 years and a coefficient of determination (*R*^2^) of 95.5%, highlighting its reliability across different age groups and its ability to capture a broader range of anatomical features compared with traditional approaches.

Similarly, Pereira de Sousa et al.^18^ assessed 554 panoramic radiographs using ML algorithms in combination with Kvaal's method. Among the tested models, the XGBoost classifier outperformed the conventional Kvaal analysis, achieving an MAE of 4.65 years compared to 5.68 years with the manual approach. One of the limitations of the study was a smaller sample size that limited the generalizability of ML models.

### Effectiveness of AI in automated age estimation based on Gustafson's criteria

Dai et al.^19^ investigated 10 ML models, based on modified Gustafson's criteria for dental age estimation in Southwest China, in their retrospective study involving 851 samples. The PLS regressor model achieved the best performance in males with an MAE of 4.151 years, while the SVR model performed well in females with an MAE of 3.806 years.

### Effectiveness of AI in automated identification of tooth, gender determination, and DVI

Milošević et al.^20^ analyzed a dataset of 86,495 tooth X-ray images using CNN models, with emphasis on sex and tooth type classification. Their findings revealed accuracies of 76.41% for sex assessment, 87.24% to 99.15% for tooth type determination, and a median absolute error of 4.94 years for age estimation. Notably, the study highlighted that models trained on healthy, unaltered teeth performed significantly better, emphasizing the impact of dental alterations (such as restorations and pathology) on predictive accuracy. Sathya and Neelaveni^21^ proposed a transfer learning approach using AlexNet for human identification based on dental traits. Their model operated in three stages: localizing the query tooth, classifying it into one of the four categories (molar, premolar, canine, incisor), and numbering it according to the universal system. This system achieved a classification accuracy of 95% across tooth types, 100% accuracy in distinguishing between upper and lower jaws, and high accuracy in numbering teeth. It surpassed traditional manual methods in both precision and speed. Fan et al.^22^ developed DENT-net, a DL system for automated human identification trained on 15,369 panoramic radiographs from 6300 individuals. This system achieved a Rank-1 identification accuracy of 85.16% and demonstrated strong performance metrics (precision 0.90, recall 0.95, *F*1 score 0.92, AUC 0.996). Additionally, its rapid processing speed (approximately 10 ms per feature extraction) makes it suitable for large-scale forensic casework. However, the system had difficulty with cases involving mixed dentition, which restricts its universal applicability. Choi et al.^23^ employed the use of AI in DVI by employing an EfficientDet-D3 CNN architecture to detect dental features on 1638 panoramic radiographs. Their model showed an average precision 99.1% and recall 99.6%. For teeth with dental prostheses the was recall 84.3% and for root canal treated teeth the recall was 89.2%, Thus the accuracy of AI models was more for natural teeth compared to those with treated teeth. Furthermore, Kim et al.^24^ explored deep neural networks for age estimation across 10,023 images, to check whether CNNs can classify dental panoramic radiographs into age groups even without precise age information, using approximate age groupings. Their study results gave an accuracy of 53.8% within ±5 years, 95.1% within ±15 years, and 99.6% within ±25 years. Also the performance was best with healthy teeth (accuracy = 96.45%), and slightly lower with treated teeth. In summary, these studies illustrate the expanding role of AI in forensic odontology beyond merely estimating chronological age. AI-driven models exhibit high accuracy in tooth classification, gender determination, and human identification, proving useful in mass disaster scenarios. However, challenges remain regarding variability due to dental alterations, population diversity, and mixed dentition, which future research should address to enhance forensic applicability.

### Comparison of various AI models used for age estimation

#### Hyperparameter-optimized deep learning models

Büyükçakır et al.^25^ tested EfficientNet, DenseNet-201, and MobileNet V3, highlighting that EfficientNet-B4 achieved the lowest MAE of 0.562 years. Their study highlighted the use of hyperparameter optimization, which substantially enhanced accuracy. Nonetheless, challenges such as limited training data and computational complexity restricted generalizability, reflecting a common barrier across dental AI studies.

#### CNNs for identification

Kim et al.^26^ employed a modified VGG16 model for human identification, which gave accuracies above 80%, despite image variability. The application of Grad-CAM visualization provided interpretability by highlighting diagnostic features. While effective, dataset imbalance and image distortion reduced reliability, emphasizing the need for data augmentation and preprocessing techniques. Bussaban et al.^27^ compared VGG19, ResNet152, and SqueezeNet across multiclass classification tasks. VGG19 consistently outperformed others, achieving 92.95% accuracy in binary classification but lower performance in more complex multiclass settings. Though EfficientNet showed promising results, smaller sample sizes constrained model learning. Heinrich^28^ trained a standardized CNN on 50,000 OPGs, achieving MAEs of 2.76 years (postmortem) and 3.26 years (antemortem), while reducing database processing times by 96%. Bizjak and Robič^29^ used DentAge, which achieved an overall MAE of 3.12 years. It gave the best results in adolescents (MAE 1.94 years), while in the elderly individuals, it did not give significant results (MAE 13.40 years), illustrating the difficulty of estimating age in older individuals. Similarly, Kahm et al.^30^ found that heuristic grouping with a tolerance of ±3 years improved *F*1 scores for WideResNet and DenseNet models.

#### Hybrid approaches

Baydogan et al.^31^ introduced Age-Net, a hybrid model integrating CNNs with ML (e.g. EfficientNetB0 + SVM). The model achieved an AUC of 0.970 and accuracy of 76.41%, demonstrating that collaborative frameworks can balance precision and strength. However, their dataset's restricted age range limited external applicability. Expanding datasets to broader age groups would enhance translational value.

#### Transfer learning and anatomical feature utilization

Mu and Gang^32^ assessed the accuracy of transfer learning models (ResNet, EfficientNet, VGGNet, DenseNet) for age estimation using panoramic radiographs. The best model was EfficientNet-B5 with an MAE of 2.8 years. The younger age group (12–21 years) gave the best results with this model. Their findings also highlight that the different anatomical structures, such as maxillary sinus and angle of the mandible, could contribute to age prediction.

Thus, after comparing the various AI models, EfficientNet variants and VGG-based models often gave the best results, with hybrid and group strategies (e.g. Age-Net) providing enhanced robustness. Dataset size and age distribution were consistently identified as key factors influencing performance, with smaller or skewed datasets leading to less generalizability. Moreover, interpretability techniques (e.g. Grad-CAM) and heuristic groupings (±3 years) have become strategies to improve clinical and forensic suitability. Collectively, these findings show that while AI models can significantly outperform traditional methods, standardizing datasets, expanding age coverage, and validating across populations are still essential for forensic adoption.

AI applications in forensics extend to various other dental fields, including periodontics, endodontics, oral medicine/pathology, prosthodontics, pediatric dentistry, orthodontics, and orofacial pain. These AI models are utilized for diagnosis, treatment planning, clinical decision-making, and prognosis prediction across different dental specialties. This technology benefits both individual patients and the community as a whole. Recently, the implementation of generative AI has emerged as a valuable tool for dental practitioners.^4,6,7^ As dental education lacks AI training, incorporating this in the postgraduate program can help to bridge the gap.^5^

## Challenges and limitations

### Data and bias

The lack of standardized data exchange frameworks and high-quality datasets is the main obstacle to AI in dentistry. Data access and sharing are also hampered by privacy and security issues. Furthermore, the fairness and generalizability of AI models can be impacted by biases in training datasets, which can result in uneven performance across demographic groups.^4^

### Ethics and accountability

Many AI systems lack transparency and explainability, which hinders accountability in clinical decision-making and erodes trust. Ethical concerns include risks to patient privacy and the potential for algorithmic bias.^4^

### Implementation barriers

The transition of AI from research to practical application is hindered by various regulatory, technical, and infrastructural challenges. Creating clinically reliable AI models requires considerable time, access to correctly labeled datasets, and substantial computational resources. Additionally, limited understanding of AI among dental professionals, along with the high costs associated with implementation and maintenance, further limit the widespread acceptance.^4^

## Conclusion

The findings of this review stress the significant potential of AI algorithms in automated age estimation processes using OPGs. The studies reviewed demonstrate that AI-based models, particularly DL architectures such as CNNs, EfficientNet, DenseNet, and hybrid models like Age-Net, exhibit superior accuracy, precision, and reliability compared to conventional age estimation techniques. These AI-driven models show promising results in reducing human error, enhancing efficiency, and improving forensic and clinical decision-making.

Despite these advancements, several challenges persist, including data scarcity, imbalanced datasets, variability in dental development across populations, and the need for standardized protocols. Although AI models have shown high performance across various age groups, further research is needed to improve generalizability across diverse populations and optimize models for forensic and clinical applications. Future studies should prioritize larger, broad-based datasets, strong validation techniques, and the integration of AI-based age estimation models into routine forensic field.

In conclusion, AI-driven age estimation using OPGs presents a transformative approach with significant forensic and clinical implications. While AI-based models may not yet fully replace traditional methods, they serve as powerful adjuncts that can enhance accuracy and efficiency. Continued research, along with the establishment of standardized AI protocols, will be essential for the widespread implementation of these models in forensic odontology and related fields.

## Abbreviations

ANN: artificial neural network
ANN-MLP: artificial neural network-multilayer perceptron
AUC: area under curve
CNN: convolutional neural network
DL: deep learning
DLM: deep learning model
DN: DenseNet
DNN: deep neural network
DVI: disaster victim identification
GBR: gradient boosting regression
mAP: mean average precision
MAE: mean absolute error
ML: machine learning
MLM: machine learning model
PLS: partial least squares
R^2^: *R*-squared value (Coefficient of Determination)
RMSE: root mean-squared error
SVM: support vector machine
SVR: support vector regressor
WRN: WiseResNet

## References

[1] Sancı A Kaya B . Applications of artificial intelligence in age estimation: a review. J Radiol Med 2024; 1: 77–83.

[2] El Joudi NA Othmani MB Bourzgui F , et al. Review of the role of artificial intelligence in dentistry: current applications and trends. Procedia Comput Sci 2022; 210: 173–180.

[3] Mohammad N Ahmad R Kurniawan A , et al. Applications of contemporary artificial intelligence technology in forensic odontology as a primary forensic identifier: a scoping review. Front Artif Intell 2022; 5: 1049584. 36561660 10.3389/frai.2022.1049584 PMC9763471

[4] Tuygunov N Samaranayake L Khurshid Z , et al. The transformative role of artificial intelligence in dentistry: a comprehensive overview part 2: the promise and perils, and the international dental federation communique. Int Dent J 2025. 10.1016/j.identj.2025.02.006 PMC11976557 40011130

[5] Dashti M Ghasemi S Khurshid Z . Integrating artificial intelligence in dental education: an urgent call for dedicated postgraduate programs. Int Dent J 2024; 74: 1466–1468. 39245622 10.1016/j.identj.2024.08.008 PMC11551563

[6] Samaranayake L Tuygunov N Schwendicke F , et al. The transformative role of artificial intelligence in dentistry: a comprehensive overview. Part 1: fundamentals of AI, and its contemporary applications in dentistry. Int Dent J 2025. 10.1016/j.identj.2025.02.005 PMC11976540 40074616

[7] Khurshid Z Waqas M Hasan S , et al. Deep learning architecture to infer Kennedy classification of partially edentulous arches using object detection techniques and piecewise annotations. Int Dent J 2025; 75: 223–235. 39645471 10.1016/j.identj.2024.11.005 PMC11806322

[8] Khurshid Z Tariq R Asiri FY , et al. Literature search strategies in dental education and research. J Taibah Univ Med Sci 2021; 16: 799–806. 34899122 10.1016/j.jtumed.2021.05.012 PMC8626813

[9] Ong SH Kim H Song JS , et al. Fully automated deep learning approach to dental development assessment in panoramic radiographs. BMC Oral Health 2024; 24: 26. 38582843 10.1186/s12903-024-04160-6 PMC10998373

[10] Sivri MB Taheri S Ercan RG , et al. Dental age estimation: a comparative study of convolutional neural network and Demirjian's method. J Forensic Leg Med 2024; 103: 102679. 38537363 10.1016/j.jflm.2024.102679

[11] Matthijs L Delande L De Tobel J , et al. Artificial intelligence and dental age estimation: development and validation of an automated stage allocation technique on all mandibular tooth types in panoramic radiographs. Int J Leg Med 2024; 138: 2469–2479. 10.1007/s00414-024-03298-w 39105781

[12] Bunyarit SS Nambiar P Naidu M , et al. Dental age estimation of Malaysian Indian children and adolescents: applicability of Chaillet and Demirjian’s modified method using an artificial neural network. Ann Hum Biol 2022; 49: 192–199. 35997704 10.1080/03014460.2022.2105396

[13] Franco A Murray J Heng D , et al. Binary decisions of artificial intelligence to classify third molar development around the legal age thresholds of 14, 16, and 18 years. Sci Rep 2024; 14: 4668. 38409354 10.1038/s41598-024-55497-5 PMC10897208

[14] Murray J Heng D Lygate A , et al. Applying artificial intelligence to determination of legal age of majority from radiographic data. Morphologie 2024; 108: 100723. 37897941 10.1016/j.morpho.2023.100723

[15] Patil V Saxena J Vineetha R , et al. Age assessment through root lengths of mandibular second and third permanent molars using machine learning and artificial neural networks. J Imaging 2023; 9: 33. 36826952 10.3390/jimaging9020033 PMC9967887

[16] Duangto P Iamaroon A Prasitwattanaseree S , et al. New models for age estimation and assessment of their accuracy using developing mandibular third molar teeth in a Thai population. Int J Leg Med 2017; 131: 559–568. 10.1007/s00414-016-1467-4 27757575

[17] Oliveira W Albuquerque Santos M Burgardt CA , et al. Estimation of human age using machine learning on panoramic radiographs for Brazilian patients. Sci Rep 2024; 14: 19689. 39181957 10.1038/s41598-024-70621-1 PMC11344797

[18] Pereira de Sousa D Diniz Lima E Souza Paulino JA , et al. Age determination on panoramic radiographs using the Kvaal method with the aid of artificial intelligence. Dentomaxillofac Radiol 2023; 52: 20220363. 36988148 10.1259/dmfr.20220363 PMC10170175

[19] Dai X Liu A Liu J , et al. Machine learning supported the modified Gustafson’s criteria for dental age estimation in southwest China. J Imaging Inf Med 2024; 37: 611–619. 10.1007/s10278-023-00956-0 PMC11031552 38343227

[20] Milošević D Vodanović M Galić I , et al. A comprehensive exploration of neural networks for forensic analysis of adult single tooth X-ray images. IEEE Access 2022; 10: 70980–71002.

[21] Sathya B Neelaveni R . Transfer learning based automatic human identification using dental traits aid to forensic odontology. J Forensic Leg Med 2020; 76: 102066. 33032205 10.1016/j.jflm.2020.102066

[22] Fan F Ke W Wu W , et al. Automatic human identification from panoramic dental radiographs using the convolutional neural network. Forensic Sci Int 2020; 314: 110416. 32721824 10.1016/j.forsciint.2020.110416

[23] Choi HR Siadari TS Kim JE , et al. Automatic detection of teeth and dental treatment patterns on dental panoramic radiographs using deep neural networks. Forensic Sci Res 2022; 7: 456–466. 36353329 10.1080/20961790.2022.2034714 PMC9639521

[24] Kim YR Choi JH Ko J , et al. Age group classification of dental radiography without precise age information using convolutional neural networks. Healthcare 2023; 11: 1068. 37107902 10.3390/healthcare11081068 PMC10137502

[25] Büyükçakır B Bertels J Claes P , et al. OPG-based dental age estimation using a data-technical exploration of deep learning techniques. J Forensic Sci 2024; 69: 919–931. 38291770 10.1111/1556-4029.15473

[26] Kim YH Ha EG Jeon KJ , et al. A fully automated method of human identification based on dental panoramic radiographs using a convolutional neural network. Dentomaxillofac Radiol 2022; 51: 20210383. 34826252 10.1259/dmfr.20210383 PMC9499198

[27] Bussaban L Boonchieng E Panyarak W , et al. Age group classification from dental panoramic radiographs using deep learning techniques. IEEE Access 2024. 10.1109/ACCESS.2024.3466953

[28] Heinrich A . Accelerating computer vision-based human identification through the integration of deep learning-based age estimation from 2 to 89 years. Sci Rep 2024; 14: 4195. 38379027 10.1038/s41598-024-54877-1 PMC10879188

[29] Bizjak Ž Robič T . DentAge: deep learning for automated age prediction using panoramic dental X-ray images. J Forensic Sci 2024; 69: 2069–2074. 39294554 10.1111/1556-4029.15629

[30] Kahm SH Kim JY Yoo S , et al. Application of the entire dental panorama image data in a artificial intelligence model for age estimation. BMC Oral Health 2023; 23: 1007. 38102578 10.1186/s12903-023-03745-x PMC10724903

[31] Baydogan MP Baybars SC Tuncer SA . Age-Net: an advanced hybrid deep learning model for age estimation using orthopantomograph images. Trait Signal 2023; 40: 1553–1563.

[32] Mu CC Gang LI . Age estimation using panoramic radiographs by transfer learning. Chin J Dent Res 2022; 25. 10.3290/j.cjdr.b3086341 35686591

